# Role of Nudt2 in Anchorage-Independent Growth and Cell Migration of Human Melanoma

**DOI:** 10.3390/ijms241310513

**Published:** 2023-06-22

**Authors:** Sana’ Hidmi, Hovav Nechushtan, Ehud Razin, Sagi Tshori

**Affiliations:** 1 Department of Biochemistry and Molecular Biology, Institute for Medical Research Israel-Canada, The Hebrew University of Jerusalem, Jerusalem 91120, Israel; sana.hidmi@mail.huji.ac.il (S.H.); sagit@ekmd.huji.ac.il (S.T.); 2 Department of Oncology, Hadassah Hebrew University Medical Center, Jerusalem 91120, Israel; hovavnech@hadassah.org.il; 3 Kaplan Medical Center, Rehovot 76100, Israel

**Keywords:** Nudt2, anchorage-independent growth, xenograft model, cell migration, melanoma

## Abstract

**Simple Summary:**

Melanoma is one of the deadliest types of skin cancer, accounting for the majority of skin cancer-related deaths. The function of Nudt2 in melanoma is unknown. The goal of this study was to examine the role of Nudt2 in melanoma cells and in vivo model. In melanoma, Nudt2 appears to be a tumor-promoting gene that could be utilized as a cancer therapy target.

**Abstract:**

Nudt2 encodes a diadenosine tetraphosphate (Ap_4_A) hydrolase that catalyzes the hydrolysis of Ap_4_A and is involved in the lysyl tRNA synthetase-Ap_4_A-Nudt2 (LysRS-Ap_4_A-Nudt2) signaling pathway. We have previously demonstrated that this pathway is active in non-small cell lung cancer. Nudt2 was shown to be involved in cell proliferation in breast cancer, making it an important target in cancer therapy. Currently, the function of Nudt2 in malignant melanoma has not been demonstrated. Therefore, we investigated the role played by Nudt2 in the growth of human melanoma. Our study showed that Nudt2 knockdown suppressed anchorage-independent growth of human melanoma cells in vitro. The in vivo effect of Nudt2 was determined by investigating the role played by Nudt2 knockdown on the ability of the cells to form tumors in a mice xenograft model. Nudt2 knockdown significantly suppressed tumor growth in this model. Moreover, overexpression of Nudt2 resulted in an increase in anchorage-independent growth of these cells, whereas Nudt2 knockdown decreased their migration. In addition, Nudt2 knockdown reduced vimentin expression. Vimentin is one of the mesenchymal markers that are involved in the epithelial mesenchymal transition (EMT) process. Thus, Nudt2 plays an important role in promoting anchorage-independent growth and cell migration in melanoma.

## 1. Introduction

**Scheme 1:**
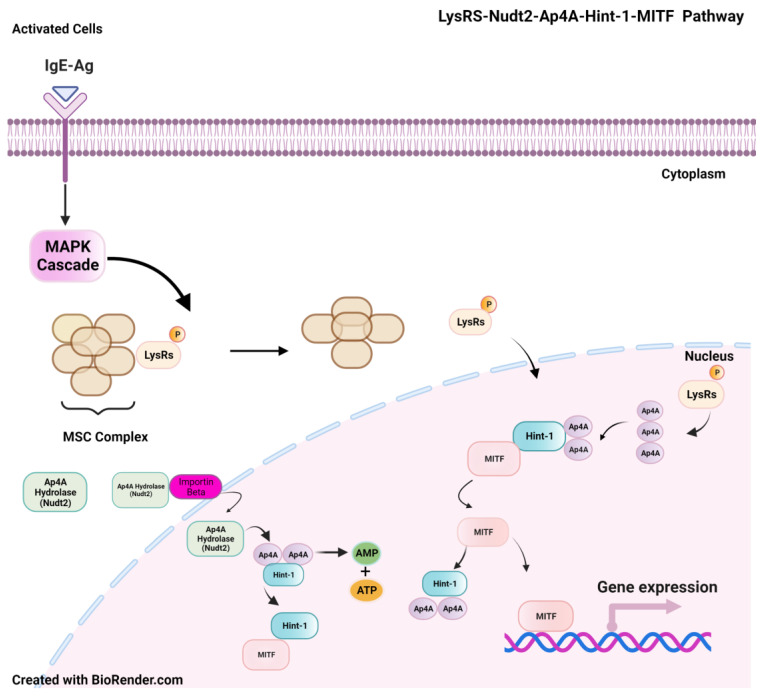
LysRS-Nudt2-Ap4A-Hint-1-MITF pathway in activated mast cells. Following mast cell activation, LysRS is phosphorylated on the serine 207 residue through the MAPK pathway. This phosphorylation dissociates LysRS from the MultiSynthetase complex (MSC), which then translocate into the nucleus. The phosphorylated form of LysRS at serine 207 can produce an Ap4A inside the nucleus. Ap4A will bind Hint-1 and MITF complex cause a dissociation of Hint-1 from MITF. Upon dissociation, MITF will be able to transcribe its target genes. Upon mast cell activation, Ap4A hydrolase (Nudt2) will associate with importin beta and it will translocate into the nucleus. The presence of Ap4A hydrolase in the nucleus leads to hydrolysis of Ap4A into AMP and ATP decreases its levels. When Ap4A level is decreased, Hint-1 will associate again with MITF, leading to the resting state in the cells.

Nudix (nucleoside diphosphate linked moiety X)-type motif 2 (Nudt2) is a member of the MutT family of nucleotide pyrophosphatases, a subset of the larger Nudix hydrolase family which is widespread among bacteria, eukaryotes, and viruses [1]. Nudt2 encodes an Ap_4_A hydrolase that is involved in the hydrolysis of Ap_4_A (diadenosine 5′, 5‴-p1, p4-tetraphosphate) to AMP and ATP, thus regulating the intracellular level of Ap_4_A. Ap_4_A is found in all living cells, both prokaryotic and eukaryotic, and studies conducted in our lab have previously identified it as a bona fide second messenger [2,3,4,5]. It is a small molecule composed of two adenosine moieties joined in a 5′-5′ linkage by a chain of four phosphates and was discovered as an in vitro synthesis product of lysyl-tRNA synthetase (LysRS) [6]. In activated eukaryotic cells, Ap_4_A is synthesized by serine 207 phosphorylated LysRS as part of the S 207 LysRS–Ap_4_A signaling pathway that was discovered and characterized by our team [3,7,8,9]. Our studies showed that in activated cells both microphthalmia transcription factor (MITF) and upstream stimulating factor 2 (USF2) are positively regulated by Ap_4_A, allowing the transcription of their respective target genes [4,5] (Scheme 1). Ap_4_A has been implicated in the function of many cellular process including apoptosis [10], DNA damage response [11], and many other signaling pathways [12,13]. Nudt2 promotes proliferation of breast carcinoma cells in vitro, and is a potent prognostic factor in human breast carcinomas, under a different mechanism of estrogen [14]. RNAseq analysis in KBM-7 chronic myelogenous leukemia cells with or without Nudt2 knockdown indicated Nudt2 involvement in cell proliferation, invasion and metastasis [15]. Knockdown of Nudt2 suppressed proliferation of several breast carcinoma cell lines by regulating mTROC1 activity via physical interaction with RagGTPases [16], suggesting that Nudt2 inhibition could have strong anti-tumor effects. Furthermore, we have shown that LysRS is phosphorylated on serine 207 in EGFR-mutated non-small cell lung cancer, and that this phosphorylation is correlated with increased disease-free survival [17]. We have also demonstrated that the LysRS-Ap_4_A pathway is active in various melanoma cell lines [2]. During the process of epithelial mesenchymal transition (EMT), epithelial markers, such as E-cadherin, will decrease and there will be an increase in mesenchymal markers, such as vimentin, N cadherin, and matrix metalloproteases (MMPs), in addition to the involvement of many transcription factors that regulate this process including snail, slug, and twist [18].

These observations indicate that the Nudt2 may have profound importance in melanoma proliferation. Thus, the main goal of the present study is to explore the role played by Nudt2 in melanoma cell function by using in vitro assays and the xenograft model. In addition, we followed the effect of Nudt2 on EMT by checking vimentin expression.

## 2. Results

### 2.1. Generation of Stable Nudt2 Knockdown Melanoma Cell Lines

**Figure 1:**
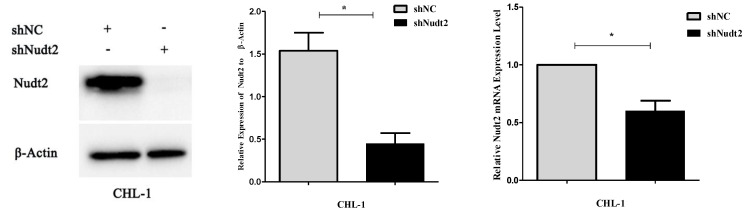
Generation of stable Nudt2 knockdown melanoma cell lines. CHL−1 melanoma cell line was infected with shRNA against Nudt2 (shNudt2) or non-targeting shRNA (shNC). Western blot analysis of Nudt2 protein in CHL−1 cell line. Relative expression level of Nudt2 to β-actin was determined via Western blot (*n* = 7). Relative mRNA expression level was determined via qPCR (*n* = 6). Results are represented as mean ± SEM. For statistical analysis, Wilcoxon signed-rank test was used. (* *p* < 0.01).

We have recently identified that the LysRS-Ap_4_A-Nudt2 pathway is involved both in non-small cell lung cancer and melanoma. Here, we investigated whether Nudt2 plays a role in melanoma cell growth by first lowering Nudt2 levels in these cells. Control and Nudt2-knocked down human melanoma cell line (CHL−1) were produced using either non-targeting or Nudt2-specific shRNA lentiviral particles. The stable knockdown of Nudt2 was verified in this cell line via the Western blot analysis and mRNA expression level was verified using real-time PCR (Figure 1 and Figure S1).

### 2.2. Nudt2 Is Required for Anchorage-Independent Growth of Melanoma Cells

**Figure 2:**
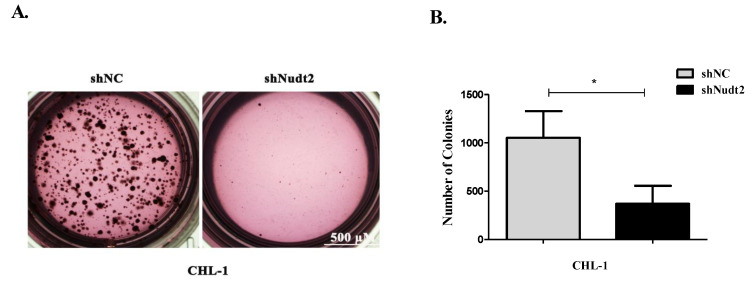
Effect of Nudt2 knockdown on anchorage-independent growth (soft agar assay). shNC and shNudt2 melanoma cells were seeded at 1 × 10^4^ in 24-well plates for 21 days. Colonies were stained with 1 mg/mL of p-iodonitrotetrazolium violet stain. (**A**). Representative photomicrographs of CHL−1. (**B**). Colonies were counted using Image J software( ij153-win-java8). The data are represented as mean ± SEM (CHL−1,*n* = 7). The two-tailed Wilcoxon signed-tank test was used for statistical analysis (CHL−1 *p* = 0.0312). (* *p* < 0.01).

Tumorigenesis and carcinogenesis require the acquisition of many tumor features, including increased cell growth rate and anchorage-independent growth potential [19]. The soft agar colony formation assay is an in vitro assay that is used to assess tumorigenesis capacity. In this assay, transformed cells gain the ability to expand in the absence of anchorage environment. The colony formation assay was used to assess the effect of Nudt2 knockdown on anchorage-independent development. Nudt2 knockdown resulted in a reduction in colony number and size CHL−1 cell line (Figure 2A). The number of colonies (Figure 2B) decreased by 88% in CHL−1 cell (*p* = 0.0312). These findings showed that Nudt2 plays an important role in promoting anchorage-independent melanoma tumor cell growth and tumorigenesis.

### 2.3. Nudt2 Knockdown Suppress Xenograft Tumor Growth In Vivo

**Figure 3:**
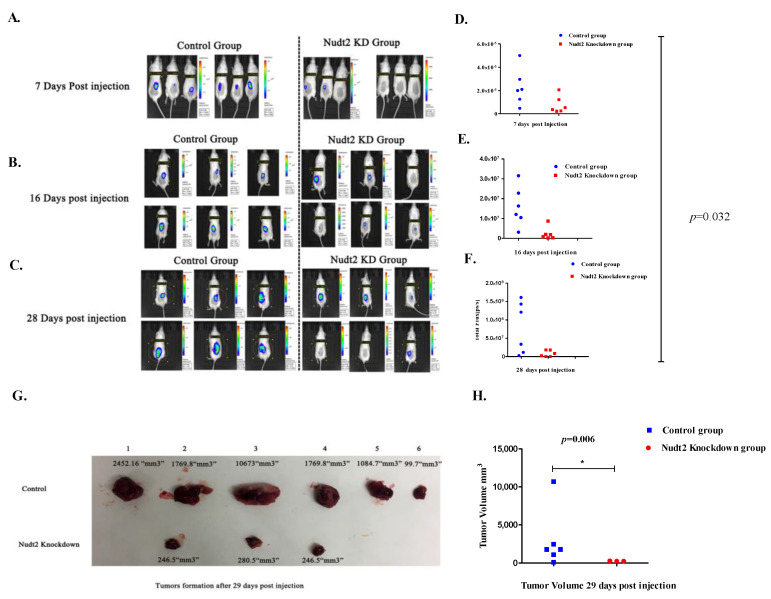
Nudt2 suppresses tumor growth in the xenograft model. CHL−1 Nudt2 knockdown or control cells were injected subcutaneously into NOD/SCID mice. (**A**–**C**). Representative images of tumor growth in bioluminescence imaging (IVIS), 7–16 and 28 days post-injections. (**D**–**F**). Representative measurement of tumor burden, 7–16 and 28 days post-injection (total Flux, photons/s), *p* = 0.032 calculated to compare total flux at day 7 vs. total flux at day 28). (**G**). Representative tumors from control group and Nudt2 knockdown group after surgical removal. (**H**). Represent tumor volume in control group vs. Nudt2 knockdown group *p* = 0. 006. Mann–Whitney test was used for the statistical analysis in the in vivo comparisons. (* *p* < 0.01).

In order to explore whether Nudt2 knockdown affects tumor growth in vivo, we used a xenograft tumor model. For this purpose, we prepared luciferase-expressing cells from Nudt2 knockdown and control cells by infecting the cells with lentiviral particles contains luciferase-expressing vector; luciferase will help us to monitor tumor growth in in vivo imaging as mentioned in detail in the Materials and Methods section. NOD/SCID mice were subcutaneously injected with either control CHL−1 melanoma cells or Nudt2 knocked down IVIS; the analysis revealed a significant difference in tumor growth between the control and Nudt2 knockdown groups (*p* = 0.032; Figure 3D,F). The tumors in mice injected with CHL−1 Nudt2 knockdown cells grew at a slower rate and had a smaller volume than the tumors in mice injected with control melanoma cells (Figure 3G,H). Tumor volume in the Nudt2 knockdown group was lower than that of the control group, with a reduction of 96%. The difference in tumor volume between the control and Nudt2 knockdown groups was significant, with a *p* value of 0.006. These results suggest that Nudt2 suppressed tumor growth in this in vivo xenograft model.

### 2.4. Nudt2 Overexpression Increased Anchorage-Independent Growth in a Melanoma Cell Line

**Figure 4:**
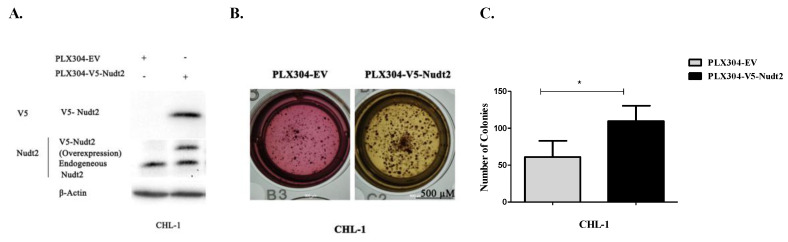
Soft agar colony formation in cells overexpressing WT Nudt2(PLX304−V5−Nudt2). Stable overexpression of WT Nudt2 was generated in CHL−1 melanoma cell and the cells were seeded at 5000 cells/250 µL in 24-well plates for 21 days. (**A**). Expression of endogenous Nudt2, overexpressed Nudt2 (V5−Nudt2) was analyzed via Western blot. (**B**). Representative photomicrographs: colonies were stained with p-iodonitrotetrazolium violet stain. (**C**). Colony numbers were counted using the Image J software(ij153-win-java8); *n* = 7. The data are represented as mean ± SEM. For statistical analysis, Wilcoxon signed-rank test was used. * *p* < 0.01.

Recent research supports Nudt2’s role in the regulation of cellular proliferation in breast cancer [14,16]. The purpose of this study was to find out whether Nudt2 has an effect on anchorage-independent growth in melanoma cell lines. To investigate this, lentiviral infection of human melanoma CHL−1 was used to produce stable cell lines overexpressing wild-type (WT) Nudt2 by using PLX304-V5-Nudt2 expressing vector. As a control, infection with an empty vector (PLX304-EV) was used. Western blot was used to compare the expression levels of WT vs. EV. V5 antibody was used to check the overexpression of Nudt2, which is tagged to V5 in the expression vector: it showed one band in overexpressed cells compared with none in control. When Nudt2 antibody was used, two bands were detected in overexpressed cells, corresponding to V5−Nudt2 fusion and to endogenous Nudt2. In control, only endogenous Nudt2 was detected (Figure 4A). The colony formation assay was used to determine the effect of WT Nudt2 overexpression (PLX304−V5−Nudt2) on anchorage-independent growth (Figure 4B and Figure S2). Overexpression of WT Nudt2 increased the colony number and colony size in CHL−1 cell compared to cells transfected with EV (PLX304−EV). These findings suggest that Nudt2 is important to support anchorage-independent growth in human melanoma cells. 

### 2.5. Nudt2 Knockdown Decreased Cell Migration

**Figure 5:**
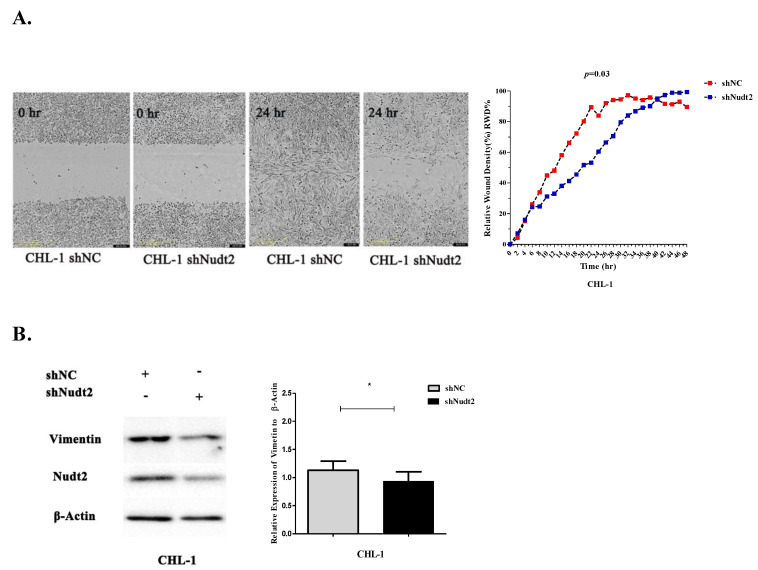
Effect of Nudt2 knockdown on cell migration. The ability of Nudt2 knockdown cells to migrate was measured in a scratch wound assay using the IncuCyte live imaging system. (**A**). Microscope imaging of migrating control (shNC) and Nudt2 knockdown (shNudt2) CH−1 cells acquired at 0 and 24 h, respectively. Relative wound density (RWD%) was measured using Incucyte software (Incucyte-2022A) metrics for every 2 h for a total of 48 h. Area under the curve (AUC) was computed by numerical integration. Data are presented as individual experiments (CHL−-1, *n* = 7). *p* values were calculated for the comparison between shNudt2 AUC and shNC AUC. (**B**). Represent expression level of vimentin in Nudt2 knockdown and control cells in Western blot. Results are represented as mean ± SEM, *p* = 0.03. (* *p* < 0.01).

Cells that have developed the ability to grow in an anchorage-independent environment will migrate and invade [20]. The aim of this study was to see if Nudt2 knockdown affected cell migration. A wound scratch model was used to test the migration of melanoma Nudt2 knockdown cell lines (Figure 5A). Every 2 h for 48 h, cell migration was calculated as a percentage of relative wound density (RWD) (Figure 5A) (*p* = 0.03). Protein expression of vimentin, a mesenchymal marker involved in EMT during metastasis, was detected in Nudt2 knockdown and control cells of CHL−1. It was observed that vimentin expression was reduced in Nudt2 knockdown cells compared to control (Figure 5B and Figure S3). Moreover, expression level of N cadherin, MMP9 and snail was checked. The results showed that there is no change in protein expression level of these proteins in Nudt2 knockdown cells compared with control (Figure S4).

## 3. Discussion

High expression levels of Nudt2 has been observed in breast cancer and it has been shown to affect cell proliferation [14,16]. In this study, Nudt2 was shown to have a significant role in promoting breast cancer proliferation by different mechanisms from estrogen [16]. Nudt2 has been demonstrated to have a role in breast cancer proliferation by regulating mTORC1 localization and activity which are regulated by Nudt2 and RagGTPase interactions [16]. This suggests that it is a tumor-promoting gene, and its high expression in breast cancer cells could make it a prognostic marker for that cancer. A previous report showed that Nudt2 knockout in chronic myelogenous leukemia cells regulate many genes involved in tumorigenesis and metastasis indicating that Nudt2 may have a major role in tumorigenic potential of cancer cells [15]. All these studies showing the importance of Nudt2 in regulation of breast cancer proliferation under different mechanisms led us to study its roles in melanoma. In this study, we found that Nudt2 knockdown inhibited anchorage-independent melanoma growth but without any effect on cell proliferation (Figure 2), and its knockdown reduced tumor growth in vivo (Figure 3). Moreover, we showed that Nudt2 overexpression increased anchorage-independent growth in human melanoma cells (Figure 4). We wanted to understand the molecular mechanism behind this effect. As previously shown, Nudt2 regulates cell proliferation in breast cancer cell lines via its effect on mTORC1 activity [16]. In this study, we investigated the effect of Nudt2 knockdown on mTROC1 downstream targets, such as p-P70 s6 kinase, but we did not see any change in these proteins’ expression level, which indicates that Nudt2 knockdown has no effect on mTROC1 activity in melanoma and the effect of Nudt2 on anchorage-independent growth in melanoma is not mediated by mTROC1 activity. Anchorage-independent growth is a hallmark of cancer and a contributor to cancer metastasis [20]. Anchorage-independent growth is connected to EMT in many cancers [21]. EMT involves many epithelial and mesenchymal markers, such as E cadherin, N cadherin, vimentin, slug, snail, and MMP9. During EMT, epithelial markers, such as E cadherin, will decrease while mesenchymal protein expression will increase [18]. In this study, we checked whether Nudt2 effect on anchorage-independent growth is accompanied by cell migration and changes in EMT markers. Our results showed that Nudt2 knockdown decreased cell migration in CHL−1and Nudt2 knockdown reduced the expression of vimentin without any change in the expression level of N-cadherin, snail, and MMP9 (Figure S4). These results indicate that Nudt2 effect on anchorage-independent growth is not fully regulated by EMT. These results confirmed that Nudt2 is a potent regulator of tumorigenicity in melanoma cell lines and in a xenograft model, indicating that Nudt2 is very important to support anchorage-independent growth in melanoma and that Nudt2 could serve as a target for cancer therapy. Further investigation is needed to understand the molecular mechanism behind the effect of Nudt2 on anchorage-independent growth in melanoma. Moreover, it is very important to determine whether Ap_4_A is involved in this process, and if it has role, what is the mechanism behind it.

## 4. Materials and Methods

### 4.1. Cell Culture

The human melanoma cell line CHL−1 (ATCC CRL-9446) was maintained in a growth medium containing DMEM (#01-052-1A, Biological Industries, Cromwell, CT, USA), 100 units/mL penicillin, 100 μg/mL streptomycin, 1 mM sodium pyruvate (#03-042-1B, Biological Industries) and 10% fetal bovine serum. 

### 4.2. Stably Transfected Melanoma Cell Lines

Human melanoma cells (1 × 10^6^) were cultured in 6-well plates to reach 60% confluence on the day of infection. They were infected with either Nudt2 shRNA lentiviral particles (#sc-60188-V; Santa Cruz Biotechnology, Almog, Israel) or control shRNA lentiviral particles (# sc-108080; Santa Cruz Biotechnology) using polybrene transfection reagent (#TR-1003-G; Merck, Herzliya, Israel) at a final concentration of 8 μg/mL. The cells were then incubated at 37 °C, in a 5% CO_2_ incubator for 24 h, after which the medium was replaced with complete medium. Another 24 h later, the medium was replaced by a puromycin selection medium, which was optimized for each cell line according to their killing curves. Selected colonies of the cells were expanded and maintained.

### 4.3. Expression Vector, Lentiviral Production, Cell Infection and Preparation of Luciferase Expressing Cells

Lentiviral production was performed using X-tremeGENE HP Transfection Reagent. A mixture of vsvg/viral envelope plasmid, dvpr/viral packaging vector, PLX304 empty vector (Addgene) or Nudt2 WT vector or PLX304-Luciferase-V5 vector were co-transfected into the 293T cells. Supernatants containing viral particles were collected and used for infection. The PLX304-Blast-V5 Nudt2 (V5-Nudt2) lentiviral expression vector (CCSB-Broad Lentiviral Expression Library, Dharmacon) was used as the expression vector. The empty PLX304 vector (pLX304 was a gift from David Root (Addgene plasmid # 25890; http://n2t.net/addgene:25890 (accessed on 6 June 2018); RRID: Addgene_25890) was used as control. CHL−1 cells was infected with either PLX304 empty vector or Nudt2 WT using polybrene transfection reagent at a final concentration of 8 μg/mL. 1 × 10^6^ cells were cultured in 6-well plates to reach 60% confluence on the day of infection. The cells were then incubated at 37 °C, in a 5% CO2 incubator for 24 h, after which the medium was replaced with complete medium. Another 24 h later, the medium was replaced by a blasticidine selection medium, which was optimized for the cell line according to the killing curve. The selected colonies of the cells were expanded and maintained. To prepare luciferase-expressing cells, 1 × 10^6^ cell of CHL−1 human melanoma stable Nudt2 knockdown and control were cultured in 6-well plates to reach 60% confluence on the day of infection. They were infected with lentiviral particles prepared using PLX304 Luciferase-V5 plasmid blast (a gift from Kevin Janes; Addgene plasmid # 98580; http://n2t.net/addgene:98580 (accessed on 19 January 2021); RRID: Addgene_98580) using polybrene transfection reagent at a final concentration of 8 μg/mL. The cells were then incubated at 37 °C, in a 5% CO_2_ incubator for 24 h, after which the medium was replaced with complete medium. Another 24 h later, the medium was replaced by a blasticidine selection medium. The selected colonies of the cells were expanded and maintained. Please see Table 1. 

### 4.4. Gel Electrophoresis and Western Blotting

Human melanoma cell lines were washed and lysed on ice with lysis buffer containing 50 mM Tris-HCl, 1% Nonidet P-40, 0.25% Na-deoxycholate, 150 mM NaCl, 1 mM EDTA, X1 protease inhibitor cocktail, 1 mM phenylmethylsulfonyl fluoride, 1 mM sodium orthovanadate, and 17 mM NaF. Proteins were resolved using 8–15% SDS-PAGE under reducing conditions and transferred to polyvinylidene difluoride membranes (Merck Millipore, Herzliya, Israel). Visualization of the proteins was performed via chemiluminescence with EZ-ECL (#20-500-1000, Biological Industries).

### 4.5. Antibodies

Antibodies against Nudt2 (#10484-1-AP, dilution 1:1000, Proteintech, Biotest, Kfar Saba, Israel), β-Actin (#A1978, dilution 1:10,000, Sigma-Aldrich, Jerusalem, Israel), V5-antibody (#v8012, dilution 1:2000, Sigma Aldrich), vimentin antibody (#5741T, dilution 1:2000, Cell Signaling, Jerusalem, Israel), N cadherin antibody (#13116, dilution 1:2000, Cell Signaling, Jerusalem, Israel), MMP9 antibody (#13667, dilution 1:2000, Cell Signaling, Jerusalem, Israel), and Snail antibody (#3879, dilution 1:2000, Cell Signaling, Jerusalem, Israel) were used. 

### 4.6. Soft Agar Colony Formation Assay

Soft agar colony formation assays were performed in 24-well plates. The lower layer was prepared from 0.8% noble agar in complete medium and 500 μL of agar; medium mixture was added to each well. In the top layer, 1 × 10^4^ cells/250 μL or 5 × 10^3^ cells/250 µL were suspended in 0.3% noble agar in complete medium. After the agar solidified, 500 μL of complete medium were added. Fresh medium was added every 3–5 days. Cells were incubated at 37 °C in a CO_2_ incubator for 21 days, after which the colonies were stained with p-iodonitrotetrazolium violet (#I8377, Sigma Aldrich, Israel ). The number of cells was counted via the Image J software(ij153-win-java8).

### 4.7. Mice

NOD.CB17-Prkdc^scid^/NCrHsd mice were purchased from Envigo Laboratory (Jerusalem, Israel). 4-week-old male mice were used in this experiment. All mice experiments were carried out under the Hebrew University’s Institutional Animal Care and Use Committee-approved protocol MD-20-15907-5. The Hebrew University is an Association for Assessment and Accreditation of Laboratory Animal Care-approved institute.

### 4.8. Tumor Xenograft Model

Mice were injected subcutaneously with CHL−1 Nudt2 knockdown or the control stable human melanoma cell line that expressed luciferase (5 × 10^6^ in 200 µL PBS). Tumor imaging was performed once a week for four weeks. For bioluminescence imaging mice, mice were injected intraperitoneally (IP) with 5 mg/mL luciferin (VivoGIo™ Luciferin, in vivo grade, #P1043, Promega, Sartorius, Beit HaEmek, Israel) in a 0.5 mL volume and anesthetized with isoflurane using a non-rebreathing system, induction 2–3%, maintenance 0.25–2%. The imaging time for mice was between 5 and 10 min. The IVIS Lumina LT system was used to capture bioluminescence images (The WOHL Institute, Translational Medicine at Hadassah Medical Center, Ein Kerem, Israel). IVIS-Lamina-X5 software (4.5.5.19626 (3 August 2017)was used to analyze all of the images. Twenty-nine days after cell injection, tumors were surgically removed from mice. The tumors were measured using a caliper and their volume was calculated using the following formula: (length × width^2^)/2. The tumors in these mice were monitored weekly using a bioluminescence imaging system (IVIS). Measurement of tumor burden via bioluminescence imaging was calculated as total flux, photons/s. 

### 4.9. Statistical Analysis

The two-tailed Wilcoxon signed-rank test was used for in vitro experiments and the Mann–Whitney test was used for in vivo study.

## Figures and Tables

**Table 1:** The lists of constructs and vectors used in this study.

#	Expression Vectors	Catalog Number/Company	Usage in This Study
1	Empty PLX304 vector (lentiviral-expressing vector)	# 25890, Addgene Plasmid	Used to prepare Control cells for overexpression system
2	PLX304-Blast-V5 Nudt2 (lentiviral-expressing vector)	CCSB-Broad Lentiviral Expression Library, Dharmacon	Used to prepare Nudt2 overexpression cells
3	PLX304 Luciferase-V5 plasmid blast (lentiviral-expressing vector)	# 98580, Addgene plasmid	Used to prepare luciferase-expressing cells to monitor tumor growth in in vivo study

## Data Availability

Supporting data can be found in the Supplementary File.

## Supplementary Materials

The supporting information can be downloaded at: https://www.mdpi.com/article/10.3390/ijms241310513/s1.
