# Supplementary material for: Role of Nudt2 in Anchorage-Independent Growth and Cell Migration of Human Melanoma
Source: Int J Mol Sci. 2023 Jun 22;24(13):10513. doi: 10.3390/ijms241310513 (PMC10341887; doi:10.3390/ijms241310513)
Supplement: Supplementary file 1 [file ijms-24-10513-s001.zip › ijms-2460696-supplementary.pdf]

Supplementary Files

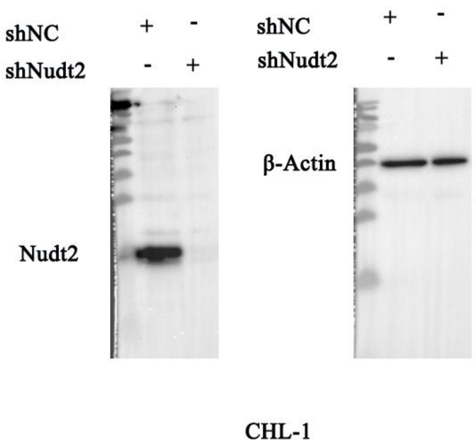

Figure S1. Original unedited blot, merged ECL images indicating Nudt2 and β-actin for representative Western blot used in Figure 1 of the manusrcipt.

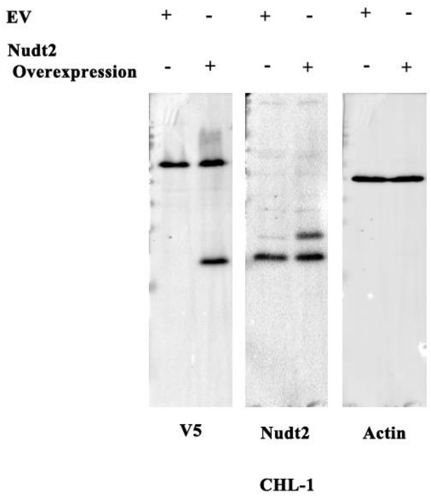

Figure S2. Original unedited blot, merged ECL images indicating V5, β-actin and Nudt2 for representative Western blot used in Figure 4A of the manusrcipt.

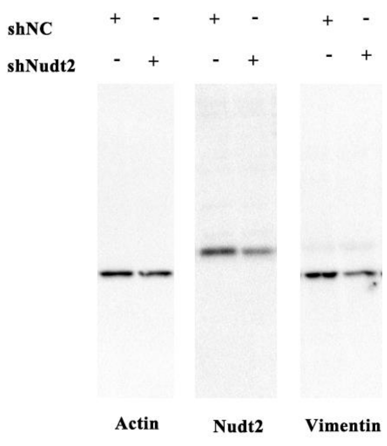

Figure S3. Original uneditted blot, merged ECL images indicating, β-actin, Nudt2 and vimentin for representative Western blot used in Figure 5B of the manusrcipt.

A.

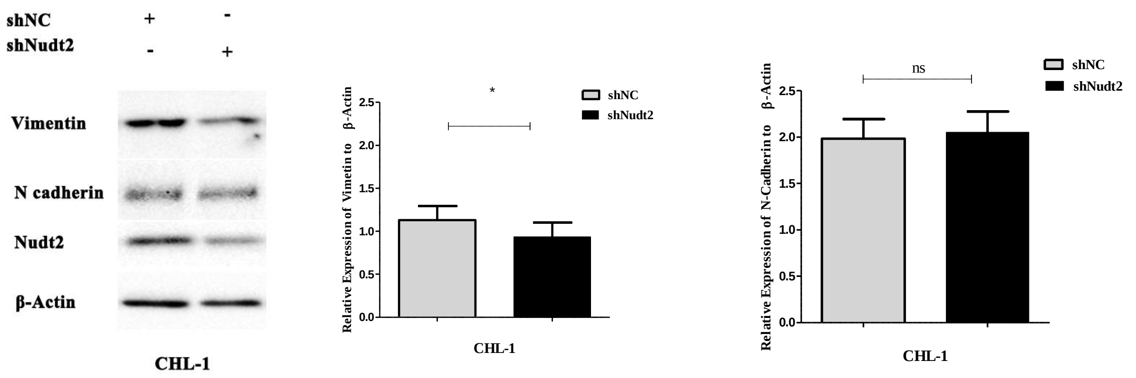

B.

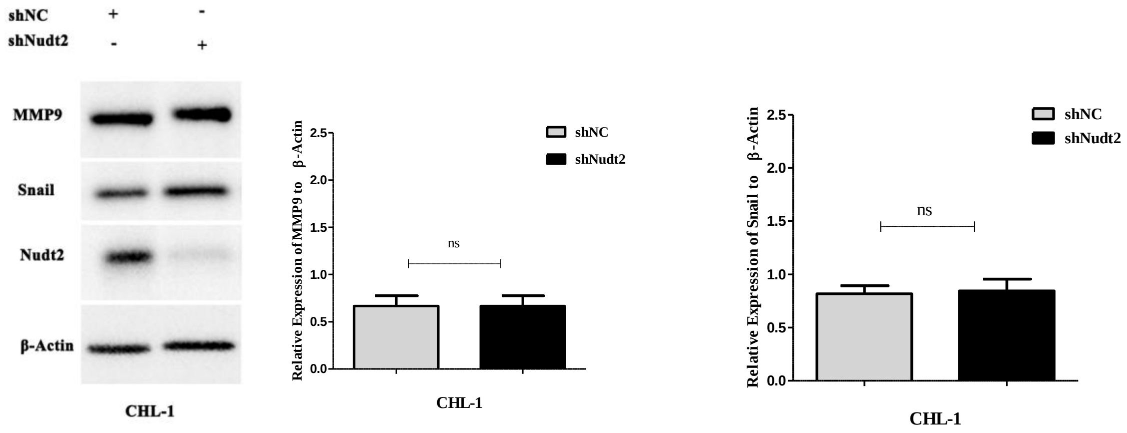

Figure S4. Effect of Nudt2 knockdown on EMT markers. (A) Represent expression level of vimentin and N cadherin in Nudt2 knockdown and control cells in Western blot. **(B)** Represent expression level of MMP9 and snail in Nudt2 knockdown cells and control cells. Results are represented as mean±SEM. (*$p < 0.01$)
